# Pneumococcal conjugate vaccine implementation in middle-income countries

**DOI:** 10.1186/s41479-017-0030-5

**Published:** 2017-03-25

**Authors:** Serena Tricarico, Hannah C. McNeil, David W. Cleary, Michael G. Head, Victor Lim, Ivan Kok Seng Yap, Chong Chun Wie, Cheng Siang Tan, Mohd Nor Norazmi, Ismail Aziah, Eddy Seong Guan Cheah, Saul N. Faust, Johanna M.C. Jefferies, Paul J. Roderick, Michael Moore, Ho Ming Yuen, Marie-Louise Newell, Nuala McGrath, C. Patrick Doncaster, Alex R. Kraaijeveld, Jeremy S. Webb, Stuart C. Clarke

**Affiliations:** 1 Faculty of Medicine, University of Southampton, Southampton, United Kingdom; 2 Institute for Life Sciences, University of Southampton, Southampton, United Kingdom; 3 University of Southampton Malaysia Campus, Johor, Malaysia; 4 Global Health Research Institute, University of Southampton, Southampton, United Kingdom; 5 International Medical University, Kuala Lumpur, Malaysia; 6 Faculty of Medicine and Health Sciences, Universiti Malaysia Sarawak, Kota Samarahan, Sarawak Malaysia; 7 Universiti Sains Malaysia Health Campus, Kelantan, Malaysia; 8 Universiti Tunku Abdul Rahman, Kampar Campus, Kampar, Perak Malaysia; 9 NIHR Southampton Respiratory Biomedical Research Unit, University Hospital Southampton NHS Foundation Trust, Southampton, United Kingdom; 10 NIHR Wellcome Trust Clinical Research Facility, University Hospital Southampton NHS Foundation Trust, Southampton, United Kingdom; 11 Faculty of Social, Human and Mathematical Sciences, University of Southampton, Southampton, United Kingdom; 12 Centre for Biological Sciences, Faculty of Natural and Environmental Sciences, University of Southampton, Southampton, United Kingdom; 13 Postal address: Infectious Disease Epidemiology Group, Mailpoint 814, Level C, Sir Henry Wellcome Laboratories, South Block, University Hospital Southampton NHS Foundation Trust, Southampton, UK SO16 6YD

**Keywords:** Immunization, *Streptococcus pneumoniae*, Pneumonia, Pneumococcal vaccines, Middle-income countries, GAVI, Health policy

## Abstract

**Background:**

Since 2000, the widespread adoption of pneumococcal conjugate vaccines (PCVs) has had a major impact in the prevention of pneumonia. Limited access to international financial support means some middle-income countries (MICs) are trailing in the widespread use of PCVs. We review the status of PCV implementation, and discuss any needs and gaps related to low levels of PCV implementation in MICs, with analysis of possible solutions to strengthen the PCV implementation process in MICs.

**Main body:**

We searched PubMed, PubMed Central, Ovid MEDLINE, and SCOPUS databases using search terms related to pneumococcal immunization, governmental health policy or programmes, and MICs. Two authors independently reviewed the full text of the references, which were assessed for eligibility using pre-defined inclusion and exclusion criteria. The search terms identified 1,165 articles and the full texts of 21 were assessed for suitability, with eight articles included in the systematic review. MICs are implementing PCVs at a slower rate than donor-funded low-income countries and wealthier developed countries. A significant difference in the uptake of PCV in lower middle-income countries (LMICs) (71%) and upper middle-income countries (UMICs) (48%) is largely due to an unsuccessful process of “graduation” of MICs from GAVI assistance, an issue that arises as countries cross the income eligibility threshold and are no longer eligible to receive the same levels of financial assistance. A lack of country-specific data on disease burden, a lack of local expertise in economic evaluation, and the cost of PCV were identified as the leading causes of the slow uptake of PCVs in MICs. Potential solutions mentioned in the reviewed papers include the use of vaccine cost-effectiveness analysis and the provision of economic evidence to strengthen decision-making, the evaluation of the burden of disease, and post-introduction surveillance to monitor vaccine impact.

**Conclusion:**

The global community needs to recognise the impediments to vaccine introduction into MICs. Improving PCV access could help decrease the incidence of pneumonia and reduce the selection pressure for pneumococcal antimicrobial resistance.

**Electronic supplementary material:**

The online version of this article (doi:10.1186/s41479-017-0030-5) contains supplementary material, which is available to authorized users.

## Background

Pneumonia is the leading infectious cause of mortality among all age groups, especially among children. It accounts for 15% of all deaths of children under five years old worldwide, and killed an estimated 922,000 children in 2015 [1]. *Streptococcus pneumoniae* is the major cause of morbidity and mortality associated with childhood bacterial pneumonia and is responsible for at least 18% of severe episodes and 33% of pneumonia deaths in children worldwide [1, 2]. It is also responsible for other invasive infections such as meningitis, sepsis and peritonitis, as well as non-invasive diseases including acute otitis media [3] with a severe burden of associated morbidity.

Since 2000, the widespread adoption of pneumococcal conjugate vaccines (PCVs) has had a major impact on the prevention of pneumonia. PCVs are projected to prevent 1 million deaths among children worldwide by 2020, and 7 million by 2030 [4]. Two conjugate vaccines are currently available: the 10-valent (PCV10) and the 13-valent (PCV13), conferring protection against ten and 13 of the most prevalent and pathogenic serotypes, respectively [5]. The most recent estimate of serotypes implicated in the global burden of pneumococcal disease in children under five years of age attributed ≥70% of the disease burden to serotypes included in both the PCV10 and PCV13 vaccines [6]. The worldwide recommendation that PCVs be included in national immunization programmes (NIPs) for children aged less than two years was renewed by the World Health Organization (WHO) in 2012, with prioritization of PCV introduction given to countries with high child mortality rates [5].

However, five of the world’s 7 billion people live in middle-income countries (MICs)^1^ [7, 8], where the majority of vaccine preventable deaths occur [7]. As of 2014, just 31% of the global target population for PCV had been immunized, with only 14 more countries adding PCV to their NIP in 2014, after it was added by 103 countries in 2013 [9]. It is the authors’ contention that in the dynamic and challenging vaccine environment, MICs may be struggling with PCV implementation without the international financial and technical support from which many low-income countries (LICs) benefit [10]. As a consequence, an opportunity to reduce a massive burden of mortality and morbidity is potentially being overlooked.

Given the number of countries where infant PCV immunization is still yet to be widely adopted, the authors undertook a systematic review into the status of PCV implementation in MICs. The review identifies potential impediments to PCV uptake and analyses possible solutions to improve PCV uptake in MICs that have yet to include PCVs in their NIP.

## Methods

### Search strategy

**Fig. 1:**
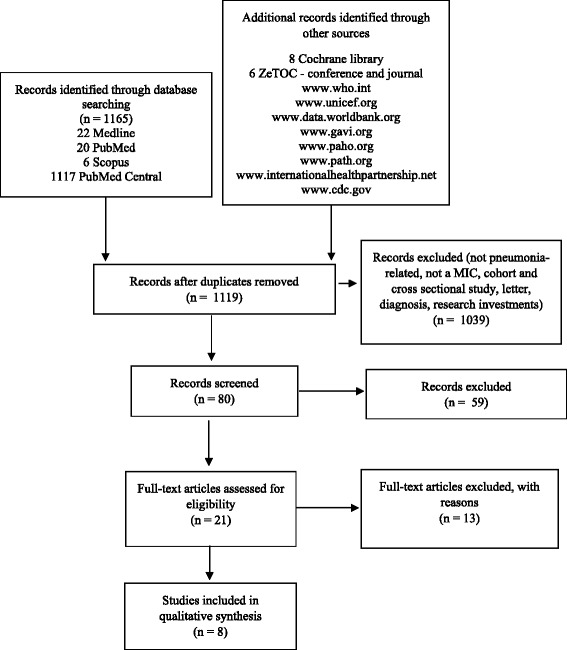
Flowchart of study selection. The following search terms were used: [*Streptococcus pneumoniae* OR pneumococcus OR pneumococcal OR pneumococci OR PCV OR pneumococcal conjugate vaccine] AND [(vaccin* adj3 policy) OR (vaccin* adj3 policies) OR (vaccin* adj3 implement*) OR (vaccin* adj3 progra*) OR (immun* adj3 progra*) OR (immun* adj3 implement*) OR (immun* adj3 policy) OR (immun* adj3 policies)] AND [middle income country OR middle income countries OR developing econom *]

Literature on the implementation of the PCV in MICs was systematically reviewed, with contributions from peer-reviewed journals and institutional websites. The following databases were searched: PubMed, PubMed Central, Ovid MEDLINE 1946, and SCOPUS. The Cochrane Library (the Cochrane Database of Systematic Reviews and the Database of Abstracts of Reviews of Effects) and Zetoc were also scanned using search terms related to pneumococcal immunization, governmental health policy or programmes, and MICs. Websites of the World Health Organization (www.who.int), the United Nations International Children's Emergency Fund (www.unicef.org), the World Bank (data.worldbank.org), the Global Alliance for Vaccines and Immunization Alliance (GAVI; www.gavi.org), the Pan American Health Organization (www.paho.org), the Program for Appropriate Technology in Health (www.path.org), the International Health Partnership (www. internationalhealthpartnership.net), Centers for Disease Control and Prevention (www.cdc.gov), the John Hopkins School of Public Health (www.jhsph.edu) and Google (www.google.com) were searched for additional data. This review was conducted according to the PRISMA statement [11] with the search filtering process illustrated in Fig. 1.

The following search terms and structure were used and modified according to the syntax requirements of the database concerned: [*Streptococcus pneumoniae* OR pneumococcus OR pneumococcal OR pneumococci OR PCV OR pneumococcal conjugate vaccine] AND [(vaccin* adj3 policy) OR (vaccin* adj3 policies) OR (vaccin* adj3 implement*) OR (vaccin* adj3 progra*) OR (immun* adj3 progra*) OR (immun* adj3 implement*) OR (immun* adj3 policy) OR (immun* adj3 policies)] AND [middle income country OR middle income countries OR developing econom*] (example of Ovid MEDLINE 1946 search syntax), with the search of terms limited to title and abstract. We found that adding specific words such as child, childhood and pediatric to the search removed useful papers, so we kept the search as wide as possible with regard to age.

### Inclusion and exclusion criteria

Two reviewers (ST and HM) performed the database search, reviewed the literature and extracted data. A continuous discussion was used as measure at all stages of the review to minimize bias and error and disagreements between the two reviewers were resolved by discussion.

SCC was regularly consulted to comment on the literature search, authoring and editorial process. The full texts of the references finally selected were assessed for eligibility using pre-defined inclusion and exclusion criteria. The search was limited to papers written in the English language, and published between 1990 and November 2015. A start date of 1990 was chosen because pneumococcal vaccines were introduced for the first time in NIP globally in the 1990s [12]. Articles reporting on active surveillance in relation to PCV policy guidance were included. Cross sectional or cohort studies, and articles relating to burden of disease, cost-effectiveness and other decision support tools and research investments (unless related to PCV policy implementation in MICs) were excluded. References were managed with the EndNote bibliographic database (Thomson Reuters, New York, United States).

## Results

The search identified 1,165 articles, of which the full texts of 80 articles were screened. The full texts of 21 were assessed for suitability (Table 1). Finally, eight papers were included in the systematic review for the qualitative analysis because they met all of the inclusion criteria (Table 1). Although the database search was performed between the years 1990 and 2015, the full-text articles assessed for eligibility were all published in the last 11 years. Seven papers were published between 2004 and 2010, and 14 papers between 2011 and 2015.

**Table 1:** Summary table of studies reporting PCV implementation in MICs

Reference	Country	Issues	Solutions
Blau et al. [14]	Albania, Azerbaijan, Georgia, Croatia	Lack of local expertise in health economic and economic evaluation. Lack of available national data on disease burden and cost of treatment of disease preventable by new vaccines.	• to use cost-effectiveness analysis to strengthen decision making in immunization policy and to ensure the sustainability of vaccine introduction • to provide economic evidence to help decide if introducing new vaccine should be prioritized along with other public health programs • to maximise the commitment and support of existing advisory bodies in the country: National Immunization Technical Advisory Group (NITAG) or Interagency Coordination Committee (ICC) • to provide scientific recommendations to support final decisions of introducing PCV
Bonner et al. [16]	LICs and MICs (list of countries not specified)	The cost of PCV can be prohibitive, discouraging countries from including it in their EPI schedules.	• GAVI and its donors should respond to WHO recommendations and countries’ needs and expand the vaccine subsidy window for vaccination in children up to age five • policy should be formulated to ensure that PCV is used in emergency contexts, including in extended age groups, as a rapid intervention to limit IPD-related morbidity and mortality • the global immunization community should address the obstacles to systematically using PCV as part of the health service package in emergencies
Gordon et al. [18]	LICs and LMICs (list of countries not specified)	Lack of economic expertise and an explicit desire to include economists in their NITAGs and interagency co-ordinating committees. The availability and consistency of financing was uniformly reported to be the greatest challenge.	
Levine et al. [20]	Global		• the need for post-introduction surveillance to monitor vaccine impact and any shifts in the serotype distribution
Moon et al. [17]	Developing countries (list of countries not specified)	No mechanism is in place ensuring that poorer countries get the lowest possible prices. This case underscores the difficulty in determining what is a “fair” price for MICs.	
Philippe et al. [13]	Global	Limited access to international support is resulting in LMICs beginning to lag behind the poorest countries in protecting their populations from vaccine-preventable diseases using newer vaccines and combination vaccines. Strong disease surveillance and programme monitoring systems are required.	• to seek more suitable formulations and presentations of new vaccines • surveillance of diseases targeted by new vaccines including enhanced laboratory networks and centres of excellence • supporting the establishment/strengthening of National Immunization Technical Advisory Committees • to ensure evidence-based decision at country level, which is particularly needed in view of the complexity of the immunization programs and cost of new vaccines
Saxenian et al. [19]	GAVI graduating countries: Indonesia, Sri Lanka, Angola, Bolivia, Azerbaijan, Honduras, Georgia, Congo, Moldova, Armenia, Mongolia, Guyana, Bhutan, Kiribati	Countries had not carried out detailed financial projections of vaccine costs by funding source.	• Ministry of Health should ensure that vaccine procurement methods result in competitive prices for high quality products • need to build specialised market knowledge and skills • a well-functioning national regulatory agencies (NRA) • to maximise the commitment and support of existing advisory bodies in the country (NITAG)
Shen et al. [15]	Developing countries (list of countries not specified)	(1) policy, standards, and guidelines; (2) governance, organization and management; (3) human resources; (4) vaccine, cold chain, and logistics management; (5) service delivery; (6) communication and community partnerships; (7) data generation and use; (8) sustainable financing. An enabling environment, even in the poorest countries, depends on the political will of decision-makers.	• a strong routine immunization platform to benefit the overall health system by generating policy and skilled human resources • NITAGs is to guide the development of national immunization policies, guidelines, and standards • NRAs are necessary if countries are to self-procure and ensure a reliable supply of quality vaccines • to improving governance, organization, and management of routine immunization include • to invest to build the capacity and professional development of an appropriately trained health care • educating and mobilising the public to support immunization and to use immunization services is central to EPI • the generation of high-quality immunization data is important to informing programmatic decisions • sustainable financing

**Fig. 2:**
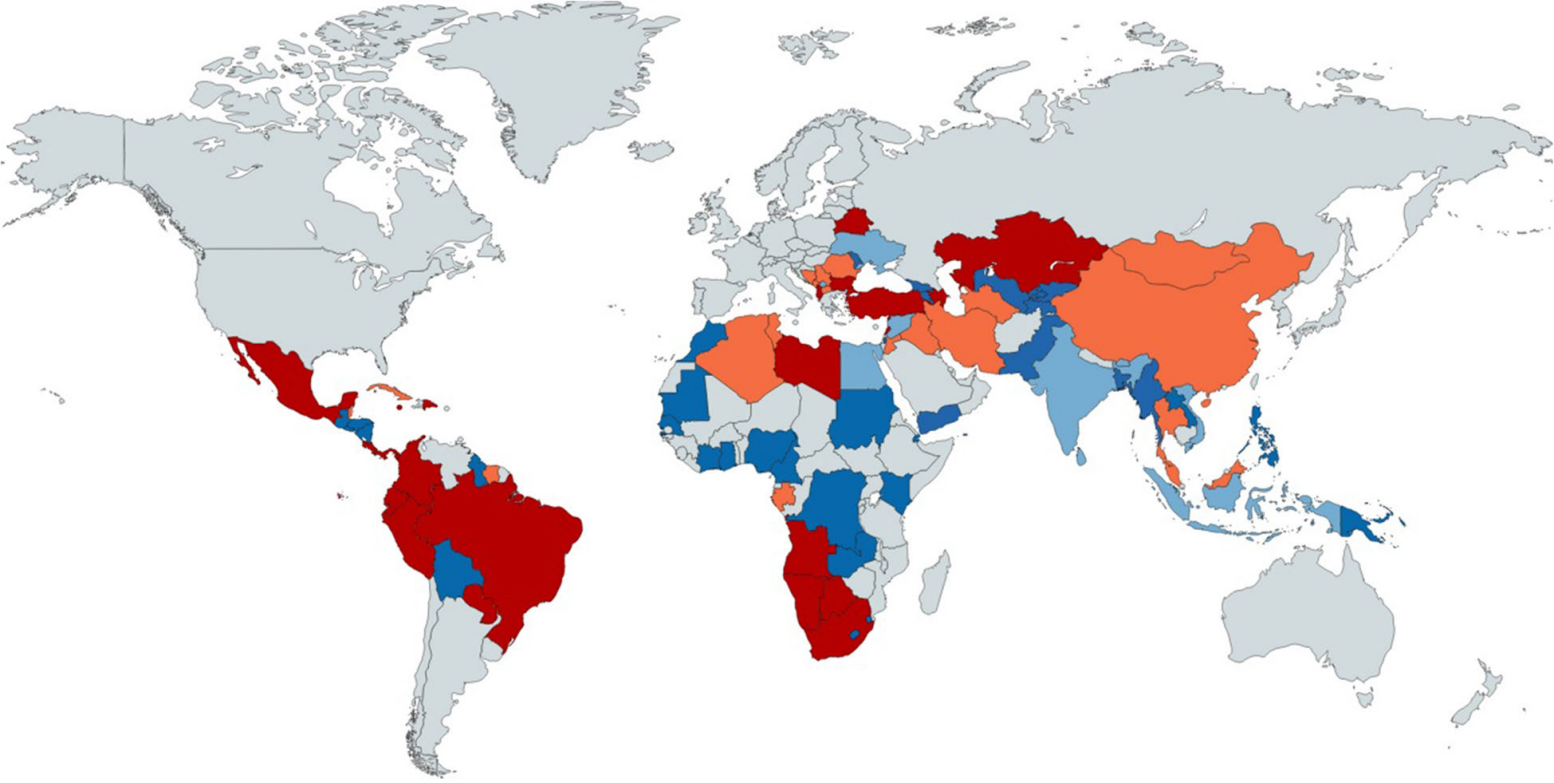
World map highlighting LMICs that have introduced PCVs in their NIP (*dark blue*), LMICs that have not yet introduced PCV in their NIP (*light blue*), UMICs that introduced PCV in their NIP (*dark red*), UMICs that have not yet introduced PCV in their NIP (*light red*). Data source: WHO/IVB Database and WorldBank, as of February 2016

Out of the 104 MICs, 43 (15/51 [29%] lower middle-income countries [LMICs] and 28/53 [52%] upper middle-income countries [UMICs]) had not introduced PCVs; 46 MICs (24 LMICs and 22 UMICs) introduced PCVs between 2000 and 2013; 13 MICs (10 LMICs and 3 UMICs) introduced PCVs between 2014 and 2015 and were supported by GAVI; 3 LMICs were approved for GAVI support in 2015 (Kyrgyzstan, Uzbekistan, and Myanmar); one graduating country^2^ (Mongolia) was approved for PCV support in 2016 and another six graduating and graduated^2^ countries have not yet applied but are eligible to do so (Bhutan, Cuba, Indonesia, Sri Lanka, Timor Leste, and Ukraine) (Table 2; Fig. 2).

**Table 2:** PCV introduction status for LMICs and UMICs

					PCV 1st dose coverage (%)							PCV 3rd dose coverage (%)							
LMIC/ UMIC	WHO REGION	Country name	PCV introduced Y/N	Year of introduction	2014	2013	2012	2011	2010	2009	2008	2014	2013	2012	2011	2010	2009	2008	Ref
UMIC	EUR	Albania	Y	3/12/2011 (Exact)	99	99	99	99	-	-	-	99	99	99	99	-	-	-	a
UMIC	AFR	Algeria	N		-	-	-	-	-	-	-	-	-	-	-	-	-	-	b
UMIC	-	American Samoa	N		-	-	-	-	-	-	-	-	-	-	-	-	-	-	b
UMIC	AFR	Angola	Y	6/3/2013 (Exact)	84	44	-	-	-	-	-	61	9	-	-	-	-	-	b
LMIC	EUR	Armenia	Y	9/15/2014 (Exact)	-	-	-	-	-	-	-	-	-	-	-	-	-	-	b
UMIC	EUR	Azerbaijan	Y	12/1/2013 (Exact)	91	5	-	-	-	-	-	64	-	-	-	-	-	-	b
LMIC	SEAR	Bangladesh	Y	3/21/2015 (Exact)	-	-	-	-	-	-	-	-	-	-	-	-	-	-	c
UMIC	EUR	Belarus	Y	2014	99	-	-	-	-	-	-	86	-	-	-	-	-	-	b
UMIC	AMR	Belize	N		-	-	-	-	-	-	-	-	-	-	-	-	-	-	b
LMIC	SEAR	Bhutan	N		-	-	-	-	-	-	-	-	-	-	-	-	-	-	b
LMIC	AMR	Bolivia (Plurinational State of)	Y	1/30/2014 (Exact)	99	5	-	-	-	-	-	56	-	-	-	-	-	-	d
UMIC	EUR	Bosnia and Herzegovina	N		-	-	-	-	-	-	-	-	-	-	-	-	-	-	b
UMIC	AFR	Botswana	Y	7/3/2012 (Exact)	99	99	99	-	-	-	-	81	65	52	-	-	-	-	b
UMIC	AMR	Brazil	Y	2010	99	-	97	99	-	-	-	93	94	88	82	24	-	-	e
UMIC	EUR	Bulgaria	Y	2010	95	96	95	96	84	-	-	92	94	94	94	69	-	-	b
LMIC	AFR	Cabo Verde	N		-	-	-	-	-	-	-	-	-	-	-	-	-	-	b
LMIC	AFR	Cameroon	Y	7/1/2011 (Exact)	93	95	92	89	-	-	-	87	88	84	70	-	-	-	b
UMIC	WPR	China	N		-	-	-	-	-	-	-	-	-	-	-	-	-	-	b
UMIC	AMR	Colombia	Y	2011	91	89	94	81	99	99	-	89	87	84	46	71	-	-	e
LMIC	AFR	Congo (the)	Y	10/11/2012 (Exact)	90	90	50	-	-	-	-	85	85	10	-	-	-	-	b
UMIC	AMR	Costa Rica	Y	2007/2008	92	-	95	-	-	-	-	83	-	89	68	97	48	-	b,e
LMIC	AFR	Côte d'Ivoire	Y	9/30/2014 (Exact)	15	-	-	-	-	-	-	2	-	-	-	-	-	-	b
UMIC	AMR	Cuba	N		-	-	-	-	-	-	-	-	-	-	-	-	-	-	b
LMIC	EMR	Djibouti	Y	12/6/2012 (Exact)	93	87	-	-	-	-	-	78	82	-	-	-	-	-	b
UMIC	AMR	Dominica	N		-	-	-	-	-	-	-	-	-	-	-	-	-	-	b
UMIC	AMR	Dominican Republic (the)	Y	2013	94	88	-	2	33	-	-	27	-	-	1	3	-	-	b
UMIC	AMR	Ecuador	Y	2010	83	86	99	99	86	17	-	99	90	94	71	17	2	-	e
LMIC	EMR	Egypt	N		-	-	-	-	-	-	-	-	-	-	-	-	-	-	b
LMIC	AMR	El Salvador	Y	2010	92	91	93	-	77	-	-	-	92	94	98	69	-	-	e
UMIC	WPR	Fiji	Y	2013	91	90	-	-	-	-	-	92	87	-	-	-	-	-	b
UMIC	AFR	Gabon	N		-	-	-	-	-	-	-	-	-	-	-	-	-	-	b
LMIC	EUR	Georgia	Y	11/24/2014 (Exact)	10	-	-	-	-	-	-	-	-	-	-	-	-	-	b
LMIC	AFR	Ghana	Y	4/26/2012 (Exact)	99	93	87	-	-	-	-	98	89	56	-	-	-	-	b
UMIC	AMR	Grenada	N		-	-	-	-	-	-	-	-	-	-	-	-	-	-	b
LMIC	AMR	Guatemala	Y	2012	78	88	3	-	-	-	-	51	24	-	-	-	-	-	e
LMIC	AMR	Guyana	Y	1/10/2011 (Exact)	98	98	94	78	-	-	-	97	96	90	50	-	-	-	e
LMIC	AMR	Honduras	Y	4/1/2011 (Exact)	86	87	88	99	-	-	-	85	87	88	78	-	-	-	e
LMIC	SEAR	India	N		-	-	-	-	-	-	-	-	-	-	-	-	-	-	b
LMIC	SEAR	Indonesia	N		-	-	-	-	-	-	-	-	-	-	-	-	-	-	b
UMIC	EMR	Iran (Islamic Republic of)	N		-	-	-	-	-	-	-	-	-	-	-	-	-	-	b
UMIC	EMR	Iraq	N		-	-	-	-	-	-	-	-	-	-	-	-	-	-	b
UMIC	AMR	Jamaica	Y	2010	48	99	-	14	-	-	-	34	70	-	17	-	-	-	b
UMIC	EMR	Jordan	N		-	-	90	-	-	-	-	-	-	-	-	-	-	-	b
UMIC	EUR	Kazakhstan	Y	2012	70	62	51	-	-	-	-	58	52	39	-	-	-	-	b
LMIC	AFR	Kenya	Y	2/14/2011 (Exact)	88	88	88	99	-	-	-	81	84	82	85	-	-	-	b
LMIC	WPR	Kiribati	Y	2013	70	62	-	-	-	-	-	57	40	-	-	-	-	-	b
LMIC	-	Kosovo	N		-	-	-	-	-	-	-	-	-	-	-	-	-	-	b
LMIC	EUR	Kyrgyzstan	Y	GAVI approved 2015	-	-	-	-	-	-	-	-	-	-	-	-	-	-	b
LMIC	WPR	Lao People's Democratic Republic (the)	Y	10/2/2013 (Exact)	79	1	-	-	-	-	-	72	-	-	-	-	-	-	b
UMIC	EMR	Lebanon	Y	2015	-	-	-	-	-	-	-	-	-	-	-	-	-	-	b
LMIC	AFR	Lesotho	Y	7/10/2015 (Exact)	-	-	-	-	-	-	-	-	-	-	-	-	-	-	b
UMIC	EMR	Libya	Y	10/1/2013 Exact	98	-	-	-	-	-	-	97	-	-	-	-	-	-	b
UMIC	EUR	Macedonia (The former Yugoslav Republic of)	N		-	-	-	-	-	-	-	-	-	-	-	-	-	-	b
UMIC	WPR	Malaysia	N		-	-	-	-	-	-	-	-	-	-	-	-	-	-	b
UMIC	SEAR	Maldives	N		-	-	-	-	-	-	-	-	-	-	-	-	-	-	b
UMIC	WPR	Marshall Islands (the)	Y	2009	70	72	83	-	98	88	-	29	22	46	-	24	49	-	b
LMIC	AFR	Mauritania	Y	11/12/2013 (Exact)	88	23	-	-	-	-	-	84	20	-	-	-	-	-	b
UMIC	AFR	Mauritius	N		-	-	-	-	-	-	-	-	-	-	-	-	-	-	b
UMIC	AMR	Mexico	Y	2008/2009	-	88	-	99	97	93	99	94	84	99	98	92	46	14	b,e
LMIC	WPR	Micronesia (Federated States of)	Y	2008	88	92	92	89	80	-	-	56	61	60	63	70	-	-	b
LMIC	EUR	Moldova	Y	10/1/2013 (Exact)	83	17	-	-	-	-	-	72	1	-	-	-	-	-	b
UMIC	WPR	Mongolia	N		-	-	-	-	-	-	-	-	-	-	-	-	-	-	b
UMIC	EUR	Montenegro	N		-	-	-	-	-	-	-	-	-	-	-	-	-	-	b
LMIC	EMR	Morocco	Y	10/20/2010 (Exact)	99	99	86	91	16	-	-	80	80	72	23	1	-	-	b
LMIC	SEAR	Myanmar	Y	GAVI approved 2015	-	-	-	-	-	-	-	-	-	-	-	-	-	-	b
UMIC	AFR	Namibia	Y	11/11/2014 (Exact)	61	-	-	-	-	-	-	-	-	-	-	-	-	-	b
LMIC	AMR	Nicaragua	Y	12/12/2010 (Exact)	99	99	99	-	-	-	-	99	99	99	91	-	-	-	b
LMIC	AFR	Nigeria	Y	12/22/2014 (Exact)	2	-	-	-	-	-	-	-	-	-	-	-	-	-	b
LMIC	EMR	Pakistan	Y	10/9/2012 (Exact)	70	65	-	-	-	-	-	70	65	-	-	-	-	-	b
UMIC	WPR	Palau	Y	2008	99	99	86	95	87	81	90	93	93	82	61	45	18	7	b
UMIC	AMR	Panama	Y	2010	98	92	99	89	99	20	-	-	-	47	63	48	4	-	e
LMIC	WPR	Papua New Guinea	Y	11/12/2013 (Exact)	-	-	-	-	-	-	-	-	-	-	-	-	-	-	b
UMIC	AMR	Paraguay	Y	2012	78	73	99	-	-	-	-	71	73	81	-	-	-	-	e
UMIC	AMR	Peru	Y	2009	99	97	99	97	95	66	-	86	85	89	82	83	9	-	e
LMIC	WPR	Philippines (the)	Y	7/17/2013 (Exact)	42	-	-	-	-	-	-	35	-	-	-	-	-	-	b
UMIC	EUR	Romania	N		-	-	-	-	-	-	-	-	-	-	-	-	-	-	b
UMIC	AMR	Saint Lucia	N		-	-	-	-	-	-	-	-	-	-	-	-	-	-	b
UMIC	AMR	Saint Vincent and the Grenadines	N		-	-	-	-	-	-	-	-	-	-	-	-	-	-	b
LMIC	WPR	Samoa	N		-	-	-	-	-	-	-	-	-	-	-	-	-	-	b
LMIC	AFR	Sao Tome and Principe	Y	11/30/2012 (Exact)	98	99	64	-	-	-	-	95	97	-	-	-	-	-	b
LMIC	AFR	Senegal	Y	11/5/2013 (Exact)	94	-	-	-	-	-	-	81	-	-	-	-	-	-	b
UMIC	EUR	Serbia	N		-	-	-	-	-	-	-	-	-	-	-	-	-	-	b
LMIC	WPR	Solomon Islands	Y	2/17/2015 (Exact)	-	-	-	-	-	-	-	-	-	-	-	-	-	-	b
UMIC	AFR	South Africa	Y	4/1/2009 (Exact)	96	-	97	98	88	45	-	90	87	98	89	64	10	-	b
LMIC	SEAR	Sri Lanka	N		-	-	-	-	-	-	-	-	-	-	-	-	-	-	b
LMIC	EMR	Sudan (the)	Y	8/1/2013 (Exact)	99	70	-	-	-	-	-	97	30	-	-	-	-	-	b
UMIC	AMR	Suriname	N		-	-	-	-	-	-	-	-	-	-	-	-	-	-	b
LMIC	AFR	Swaziland	Y	4/23/2014 (Exact)	90	-	-	-	-	-	-	89	-	-	-	-	-	-	b
LMIC	EMR	Syrian Arab Republic (the)	N		-	-	-	-	-	-	-	-	-	-	-	-	-	-	b
LMIC	EUR	Tajikistan	Y	2015	-	-	-	-	-	-	-	-	-	-	-	-	-	-	b
UMIC	SEAR	Thailand	N		-	-	-	-	-	-	-	-	-	-	-	-	-	-	b
LMIC	SEAR	Timor-Leste	N		-	-	-	-	-	-	-	-	-	-	-	-	-	-	b
UMIC	WPR	Tonga	N		-	-	-	-	-	-	-	-	-	-	-	-	-	-	b
UMIC	EMR	Tunisia	N		-	-	-	-	-	-	-	-	-	-	-	-	-	-	b
UMIC	EUR	Turkey	Y	2008	96	97	97	98	-	-	-	96	97	97	96	93	97	-	b
UMIC	EUR	Turkmenistan	N		-	-	-	-	-	-	-	-	-	-	-	-	-	-	b
UMIC	WPR	Tuvalu	N		-	-	-	-	-	-	-	-	-	-	-	-	-	-	b
LMIC	EUR	Ukraine	N		-	-	-	-	-	-	-	-	-	-	-	-	-	-	b
LMIC	EUR	Uzbekistan	Y	GAVI approved 2015	-	-	-	-	-	-	-	-	-	-	-	-	-	-	b
LMIC	WPR	Vanuatu	N		-	-	-	-	-	-	-	-	-	-	-	-	-	-	b
LMIC	WPR	Viet Nam	N		-	-	-	-	-	-	-	-	-	-	-	-	-	-	b
LMIC	-	West Bank and Gaza	N		-	-	-	-	-	-	-	-	-	-	-	-	-	-	b
LMIC	EMR	Yemen	Y	1/29/2011 (Exact)	93	94	88	81	-	-	-	88	88	82	56	-	-	-	b
LMIC	AFR	Zambia	Y	5/10/2013 (Exact)	89	-	-	-	-	-	-	77	-	-	-	-	-	-	b

Sources: a, [12]; b, [46]; c, [47]; d, [48]; e, [49]

A lack of country-specific data on disease burden is considered one of the leading causes of delay in PCV implementation, particularly in relation to the burden of pneumonia and other acute respiratory tract infections [13–15] (Table 1). The prohibitive cost of PCV is discouraging countries from including it in their NIPs [13, 15–18] and a lack of local expertise in economic evaluation [14, 18, 19] was also identified as a recurring problem (Table 1). Thus, the commonly suggested solutions to combatting the underuse of PCVs were the use of cost-effectiveness analysis and the provision of economic evidence to strengthen decision making in immunization policy [13–15, 18], the evaluation of the burden of disease with pre-assessments, and post-introduction surveillance to monitor vaccine impact and any shifts in the serotype distribution [13, 15, 18, 20] (Table 1). Maximizing the commitment and support of existing advisory bodies in MICs, national immunization technical advisory groups (NITAGs) or interagency coordination committees (ICCs) to provide scientific recommendations to support final decisions of introducing PCV [13–15, 18, 19] was also recommended, along with the expansion of the vaccine subsidy window by GAVI and its donors, in order to respond to WHO recommendations and countries’ needs [16] (Table 1).

## Discussion

This systematic review provides an update on the status of, and impediments to, PCV implementation in MICs. Although PCVs have been available since 2000, the literature assessing the problems MICs experience in implementing widespread PCV immunization has only been published since 2008. This review found that there has been some progress since 2013, but most MICs have not yet added PCVs to their NIPs for infants.

The significant difference in the uptake of PCV in LMICs and UMICs, 71% and 48% respectively, is mainly due to an unsuccessful process of ‘graduation’of MICs. Once a country crosses the income eligibility threshold for vaccine subsidy support by GAVI, the financial assistance phases out in a ‘graduation’ process. GAVI’s graduation process is designed to ramp up domestic co-financing of vaccines; however, once GAVI support ends, the new UMICs may not be able to fully fund these vaccines.

Our review has found that a lack of country-specific data on disease burden is considered one of the leading causes of delay in PCV implementation in UMICs, together with a lack of local expertise in economic evaluation, and the cost of PCV. While WHO recommends that PCV be used, despite the lack of country-specific pneumococcal surveillance data [5], PCV is one of the most expensive vaccines that WHO recommends for inclusion in NIP. The cost can be prohibitive, discouraging many MICs from including it. PCV is sold at USD$3.30–$7 per dose (when purchased through GAVI); USD$14.12–$15.68 per dose to the Pan American Health Organization (PAHO) Revolving Fund [4, 21], and US$159.58 per dose to the private sector (pediatric PCV13) [22]. Therefore, in order to fully fund their immunization programmes, MICs should improve informed decision-making on vaccine introduction and other areas of immunization policy and enhance national funding of immunization through advocacy, technical assistance, and training.

The two most populated countries in the world, China and India, require an important mention. China (1.371 billion people) and India (1.311 billion people) [23] are still classified as LMICs and have not added PCV to their NIP. This means that, excluding the unmeasurable percentage of people who received PCV privately, almost 36.4% of the entire world population has not received PCV (See Additional file 1). The Indian government recently announced the possible introduction of PCV in a phased manner by 2017–18 [24, 25]. Mainland China has not yet included PCV in its publicly funded Expanded Program on Immunization (EPI), but it is available at immunization clinics for a fee [26]. However, Hong Kong did add PCV to its NIP in 2009. Without actions in these priority areas, a likely substantial reduction of child mortality and morbidity from pneumonia will not be reached.

Although substantial achievements have been made in preceding decades with other immunization programmes in MICs (e.g. diphtheria-tetanus-pertussis [DTP3], *Haemophilus influenzae* type b [Hib]) [27], PCV implementation is still lagging behind in these countries [28]. A similar delay in implementation has been observed for five other priority new or underused vaccines, namely rotavirus, human papilloma virus [HPV], inactivated poliovirus vaccine [IPV], Japanese encephalitis, and yellow fever vaccine. According to the latest WHO estimates, this leaves 20% of MICs unprotected from these important pathogens, [7]. The main issue is that the majority (73%) of the world’s poor people (defined as people living at or below US$1.90 a day [8]) now reside in MICs, which also have the highest rates of vaccine preventable deaths [7]. MICs are home to five of the world’s 7 billion people yet (with donors focused on assisting LICs) they have been slow to introduce PCVs. This results in a missed opportunity to dramatically reduce avoidable morbidity and mortality [7, 8].

### Overview of PCV procurement opportunities

For the period 2010–2015, GAVI committed approximately US$1.9 billion through the pneumococcal Advance Market Commitment (AMC) to fund PCVs that are suitable for developing countries [29]. Those countries graduating or who have graduated from GAVI support and who have not yet been approved for PCV are able to apply for subsidized PCVs, with the terms and conditions of AMC set at a maximum of US$3.50 per dose [4]. Alongside this, the International Finance Facility for Immunization (IFFIm) provided US$41.58 million toward GAVI’s PCV programme in 2011, with the aim to immunize more than 3 million children and prevent more than 1.5 million deaths by 2020 [30]. Prior to this, GAVI established the Accelerated Vaccine Introduction (AVI) initiative in 2008 with the core goal to broaden and speed up access to PCVs over the period 2009–2015 [31].

The WHO has actively developed different programmes to help MICs with PCV implementation. The WHO Integrated Global Action Plan for the Prevention and Control of Pneumonia and Diarrhoea (GAPPD) aims to reduce deaths from pneumonia to fewer than 3 children per 1,000 live births by 2025 [32]. The WHO Expanded Programme of Immunization has seen a dramatic increase in the implementation of new and under-utilized vaccines providing additional prevention of untimely deaths and disabilities, including from pneumococcal disease [33].

The PAHO Revolving Fund, also known as the Regional Revolving Fund for Strategic Public Health Supplies, helps Latin American countries negotiate a lower cost of PCV through bulk procurement, technical assistance on supply management, and assistance with planning, procurement systems, warehousing and distribution, and quality assurance [21]. PAHO’s hospital-based surveillance network for bacterial pneumonia currently includes 10 MICs of which five are LMICs (Bolivia, El Salvador, Guatemala, Honduras, and Nicaragua) and five are UMICs (Brazil, Ecuador, Panama, Paraguay, and Peru) [21].

The WHO MIC Task Force has committed to investing approximately US$20 million per year for the 2016–2020 period to support activities included in the MIC Strategy [7]. It includes strengthened decision-making for timely and evidence-based immunization policy, increased political commitment and financial sustainability of NIPs, enhanced demand for and equitable delivery of immunization services, and improved access to affordable and timely supply.

The WHO Strategic Advisory Group of Experts on Immunization (SAGE) Global Vaccine Action Plan (GVAP) aims to make 2011–2020 the ‘Decade of Vaccines’ [34]. Its target was to introduce at least one under-utilized vaccine by 2015 into 90 LICs and MICs. So far, PCV has been the most frequently introduced vaccine. An estimated US$42 billion to US$51 billion will go towards expanding access to routine immunizations and introducing additional vaccines to routine immunization programmes.

The international non-profit organization Program for Appropriate Technology in Health (PATH) is collaborating with private- and public-sector partners to advance the development of PCVs in LICs [35]. In particular, their vaccine development portfolio includes projects to advance protein vaccines that can provide broad, affordable protection across the many varieties of the pneumococcus, as well as PCVs that are tailored to the health and cost needs of low-resource countries.

In 2015, Doctors Without Borders/Médecins Sans Frontières (MSF) launched a global campaign—‘A Fair Shot’— calling on GlaxoSmithKline (GSK) and Pfizer to slash the price of PCV in developing countries, including MICs, to US$5 per child so that more children can be protected, and to disclose what they currently charge countries for the vaccine [36]. MSF believes that governments supporting GAVI must pressure companies to disclose the price they charge for the PCVs in all countries.

### Solutions

With many initiatives aimed at improving access to vaccines predominantly being targeted only at LICs, MICs have a much slower rate of PCV uptake [7]. The inclusion of PCV in MIC NIPs is also less widespread than in developed countries. This is due to cost and poor knowledge of the burden of pneumococcal disease in MICs, as well as little logistical and technical support for MICs on how to formulate and implement a coherent policy on PCV immunization. MICs need assistance in integrating PCV immunization into their health systems. Greater political commitment is required towards commissioning epidemiological studies of pneumococcal disease and subsequent resource mobilization towards widespread PCV use at a national policy level. Carriage studies and disease surveillance of *S. pneumoniae*, including disease burden and cost-effectiveness analyses, will generate the data needed to define the economic saving of widespread PCV implementation and to monitor the ongoing impact of widespread PCV immunization at national level.

The first consequence of PCVs being licensed for use and yet not being added to the NIP is that there may be substantial use of PCV in the private sector. This creates a gap between richer and poorer classes with a consequent equity issue, especially since the poorest children tend to experience the highest disease burden. Disparities in access to vaccines are often poorly understood by decision makers, particularly in licensure or in implementation strategies for new vaccines. It is therefore important to apply pressure at national and regional level to ensure governments attempt to address these equity issues to help reduce child morbidity and mortality from pneumonia.

Since the cost per dose for PCVs is among the highest of the routine childhood immunizations, enhanced international advocacy on behalf of MICs for greater flexibility on pricing, and more rational procurement mechanisms for PCVs are critical actions. In particular, the lack of competition among manufacturers is a substantial barrier to reduction of vaccine cost. In fact, only two manufacturers, Pfizer and GSK, license and produce the two currently used PCVs—PCV13 and PCV10, respectively. Moreover, currently 60% of all GAVI-procured vaccines are manufactured in India. Through a recent partnership, GAVI and the Government of India will work together to create a more sustainable vaccine manufacturing base within India, ensuring valuable supplies for the children living in all 72 other GAVI-supported countries [25]. International donors should be encouraged to provide assistance to developing country manufacturers to produce vaccine nationally. Also, ‘pooled procurement’, which combines several buyers into a single entity that purchases vaccines on their behalf (generally at lower price per dose), would help vaccine procurement for PCV introduction in MIC [37]. Comprehensive multi-year strategic plans for immunization including PCV should be developed by all MICs, and NITAGs (or equivalent committee types) should be established—their role was found to be key in LMICs adoption of PCVs [38]. Alongside these, the World Bank Country Procurement Assessment Report is intended to be an analytical tool to evaluate the existing health system of a country, and may be useful in devising vaccine priorities.

In recent years, the anti-vaccination movement has become more vocal and even hostile [39, 40]. It is a matter of concern that some researchers from the anti-vaccine movements who can influence policy have advocated against the use of available technology due to perceived risks, often without scientific evidence. International organizations (e.g. WHO) and governments, particularly in LMICs, should ensure the implementation of evidence-based public practice. Only standard surveillance systems and research conducted by public health researchers can provide appropriate evidence for decision-making.

It is in the interests of the international community to be more aware of the immunization issues faced by many MICs. Uneven global uptake of PCVs will affect serotype dynamics and spread of antimicrobial resistance, which will have an impact beyond the borders of countries without widespread PCV immunization. Levels of resistance in *S. pneumoniae* are of international concern and were noted in a 2013 ‘Threat report’ issued by the US Center for Disease Prevention and Control (CDC) [41] and there has been increasing resistance across Asia [42]. There have also been observed decreases in prevalence of *S. pneumoniae-*related resistance after implementation of PCV programmes [43, 44].

Donors and non-governmental organizations (NGOs) can contribute towards building capacity in public health surveillance by offering technical assistance in strategy and execution of widespread epidemiological surveillance towards informing immunization policies. This is crucial as the dynamics of pneumococcal epidemiology are complex and studies need to be well designed and the data properly analysed to optimize the quality of data that eventually feeds through to informing vaccine policy.

Additionally, MICs should be supported in identifying and initiating discussions with regional and local organizations with expertize in the planning, logistics and training required before implementing PCVs into a NIP. A successful example of this support model is the PAHO Revolving Fund (described earlier), which assists Latin American countries in managing the range of activities required for the implementation of PCVs.

To address and resolve the issue of poor implementation of PCVs in MICs, the authors suggest that MICs need to be considered as a whole group and collectively undertake a series of steps. MICs should undertake a mapping exercise of their procurement strategies and range of practices; build a central headquarters to collaborate with single countries; understand procurement challenges and opportunities; develop a structural framework for MICs to assess their own procurement systems; explore inter-country and pooled procurement mechanisms; and improve targeted collaborations between MICs and international funding organizations.

## Limitations

A number of study constraints exist. First, there are a limited number of studies examining PCV policy. Second, the definition of MICs as a category is sometimes somewhat arbitrary, making analysis of the MIC data difficult. Gaps also exist in the available literature, which combined heterogeneous studies. The methodology used and the article types of the selected papers were also heterogeneous, so that very few quantitative data can be extracted from literature. The study examined grey literature for evidence of unpublished data or studies, but, given the complexity of grey literature, publication bias can be present. There are also many consultations, national and regional meetings and projects (e.g. The Pneumococcal Awareness Council of Experts [PACE] [45]) which are held to discuss new vaccine introductions, but whose proceedings are often not published.

## Conclusions

The MICs are slowly implementing PCVs. The global community needs to recognise the barriers to PCV use in many MICs and respond to the situation by increasing scientific, financial, procurement and logistical support to broaden PCV access in MICs. MICs themselves need to strengthen decision making on pneumococcal immunization policy and to mobilize national political will and financing to reduce a significant, largely preventable disease burden, for the benefit of their populations and in the interests of wider international public health.

## Additional file

Additional file 1: PCV immunisation coverage in MICs. (XLSX 35 kb)

## Abbreviations

AMC: Advance market commitment
AVI: Accelerated vaccine introduction
CDC: Center for Disease Prevention and Control
DTP3: Diphtheria-tetanus-pertussis
GAPPD: Integrated Global Action Plan for the Prevention and Control of Pneumonia and Diarrhoea
GAVI: Global Alliance for Vaccines and Immunization Alliance
GSK: GlaxoSmithKline
GVAP: Global Vaccine Action Plan
Hib: *Haemophilus influenzae* type B
ICC: Interagency Coordination Committee
IFFIm: International Finance Facility for Immunization
LIC: Low-income country
LMIC: Lower middle-income country
MIC: Middle-income country
MSF: Médecins Sans Frontières/Doctors Without Borders
NGO: Non-governmental organization
NIP: National immunization programme
NITAG: National Immunization Technical Advisory Group
PAHO: Pan American Health Organization
PATH: Program for Appropriate Technology in Health
PCV: Pneumococcal conjugate vaccine
SAGE: Strategic Advisory Group of Experts on Immunization
UMIC: Upper middle-income country
WHO: World Health Organization

## Footnotes

1 As of 1 July 2015, middle-income countries (MICs) are defined as those with a Gross National Income (GNI) per capita (calculated using the World Bank Atlas method) of more than US$ 1,045, but less than US$ 12,736 ([50]. WorldBank. 2015 [Accessed 2015 November]. Available from: http://data.worldbank.org/news/new-country-classifications-2015). Of the 104 MICs, 51 are lower-middle-income (LMICs) and 53 upper-middle-income countries (UMICs), separated at a GNI per capita of US$ 4,125 ([50]. Ibid.). In the dynamic and challenging vaccine environment, MICs face increasing technical and economic issues to maintain levels of vaccine introduction and implementation comparable with lower-income countries (LICs) that benefit from international financial and technical support ([10]. Global Alliance on Vaccination and Immunization. 2015. Available from: http://www.gavi.org/support/nvs/pneumococcal/).

2 In November 2013, the GAVI Board agreed to strengthen GAVI’s approach to transition (formerly referred to as “graduation”) to support countries in the accelerated transition phase. Each year, some countries enter the accelerated transition phase and start phasing out from GAVI support, as their GNI per capita on average over the previous three years increases beyond the eligibility threshold (set at US$1,580 in 2015) [51]. GAVI. 2016. Available from: http://www.gavi.org/support/apply/graduating-countries/.
